# DNA-triplex Forming Purine Repeat Containing Genes in *Acinetobacter baumannii* and Their Association with Infection and Adaptation

**DOI:** 10.3389/fcimb.2017.00250

**Published:** 2017-06-16

**Authors:** Himanshu N. Singh, Moganty R. Rajeswari

**Affiliations:** Department of Biochemistry, All India Institute of Medical SciencesNew Delhi, India

**Keywords:** *Acinetobacter baumannii*, DNA-triplex, purine repeat, multi-drug resistant, bioinformatics analysis, genome sequence analysis

## Background

*Acinetobacter baumannii* (*A. baumannii*) is a Gram-negative, coccobacilli aerobic pathogen which causes nosocomial infections in hospitals worldwide. *A. baumannii* has been identified as one of the six important and highly drug resistant hospital pathogens by the “Infectious Disease Society of America (IDSA)” (Talbot et al., 2006; Boucher et al., 2009). *A. baumannii* rapidly acquired antibiotic resistance, leading to wide-spread multidrug-resistant isolates, which are impervious to nearly all antibiotic treatments (Fournier et al., 2006; Vashist and Rajeswari, 2006; Perez et al., 2007; Roca et al., 2012). Therefore, dissection of the molecular details at gene level of *A. baumannii* biology is urgently needed which can greatly help in our understanding of the resistance mechanisms that are evolved by the pathogen. Recently, some efforts were made to understand the relation between junk DNA, mutation frequencies with respect to antibiotic resistance in bacteria (Martinez and Baquero, 2000; Gil and Latorre, 2012) and in *A. baumannii* (Tiwari et al., 2012). However, very little is known about the role of specific virulence factors and cellular processes that are associated with the development of resistance in *A. baumannii*. A small fraction of genomic DNA is known to adopt non-canonical B-DNA (unusual) structures, and can cause genomic instability. This fact is well known in eukaryotes, however, is also suggested that it can apply in bacteria also (Martín-Lozano et al., 2002). In this connection, the purine repeat sequences attract special attention due to their high ability to convert the double stranded DNA structures to triple stranded DNA structures. The poly(purine) tracts by forming DNA triplex structures are prone to induce mutations as well as interventions in various cellular processes including replication, transcription etc. (Wells, 2008; Singh and Rajeswari, 2015). Therefore, it is interesting to look at the genes containing purine repeats in bacterial infection and in resistance and as an example, we performed a systematic search in susceptible and drug resistant strains of A. baumannii for exclusively poly(purines).

It is important to mention that the DNA triplex was first proposed by Pauling and Corey (1953) which was later proved to exist in solution by Felsenfeld et al. (1957). DNA-triplex structure, as the name suggests, is formed between three strands of DNA, the third strand forms Hoogsteen base-pairs with that strand which is rich in purines; while the Watson-Crick basepairs remains intact. DNA triplexes could be of either intra-molecular where the third strand is part of the DNA or inter-molecular if the third strand is from external DNA. Further, DNA-triplex is known as “Purine motif (R)” if the third strand is rich in purines, alternately it is called “Pyrimidine motif (Y)” where the third strand contains largely pyrimidines (Lyamichev et al., 1986; Frank-Kamenetskii and Mirkin, 1995; Rajeswari, 2012; Dyke, 2013).

It has been reported that purine repeats have potential to form DNA-triplex which can induce mutation and/or genetic instability which can further influence DNA replication, repair and transcription in eukaryotes (Bacolla et al., 2004, 2006; Jain et al., 2008). Keeping in view of the above discussion on the importance of purine repeats in causing genomic instability via DNA-triplex formation, we investigated the whole genome of *A. baumannii* to see if there are any potential purine repeat sequences which can form intra-molecular DNA triplex in causing drug resistance.

## Materials and methods

### Genome information

Genome sequence in FASTA format and annotation information in GenBank format of all the 14 strains of the *Acinetobacter baumannii* which include susceptible (SC) and multi-drug resistant (MDR) strains (Accession Number of strains are given in Table 1) were retrieved from the NCBI Nucleotide browser (https://www.ncbi.nlm.nih.gov/nuccore) on February, 2016.

**Table 1:** Distribution and enrichment of purine repeats and characteristics of DNA-triplex formed by purine (*n* > 20) repeats; and their associated genes in various strains of *Acinetobacter baumannii*.

**Bacterial strains (Accession number)**	**Bacterial genome size (Mb)**	**Purine repeat density in genome**		**PR carrying gene**	**Length**	**PR Sequence (Intra-molecular triplex motif)**	**Score (*p*-Value)**	**Tm (°C)**
		Frequency in genome (N)/Copy number per Mb in genome (^*^D_N_)	Sum total of PRs length in genome (L)/PR length per Mb in genome (^**^D_L_)					
**SUSCEPTIBLE STRAINS (SCS)**								
ATCC-17978 (CP000521.1)	3.98	79 /19.85	1295/325	Hemerythrin-like metal-binding domain-containing protein	29	AGAGAAAAAGAAAAGA**AGAAAGGAAA** GAA	18 (0.0001)	47.3
				Intergenic	22	GG **AAAAAGGA** AAGAAGGAAAAA	16 (0.0005)	31.5
						GGAAAAAGGAAAGA **AGGAAAAA**	16 (0.0005)	31.5
				Intergenic	21	GAGAAAAAGGAAAGGAGAGGA	None	
AB307-0294 (CP001172.1)	3.76	56 /14.89	905/241	Adenylate cyclase 1	22	AAAAAAAGAAAAA **AAGGAGGAA**	15 (0.0012)	33.6
				ABC1 family protein	21	**GAAGAAGAA** AAAGAGAAGAAG	17 (0.0002)	51.4
						GAAGAAGAAAAA**GAGAAGAAG**	16 (0.0005)	51.4
SDF (CU468230)	3.42	59 /17.25	964/282	Intergenic	25	GAGAAAAGGAAAGGA**GAGGAGAGG** A	16 (0.0006)	60.4
						GAGAAAAGGAA **AGGAGAGGAG** AGGA	17 (0.0002)	61.4
				Fragment of putative nitrate transporter transmembrane protein (MFS super family) (part1)	21	GGAAGAAAAGAAAAGAAAAGA	None	
**MULTIPLE DRUG RESISTANT STRAINS (MDRS)**								
AB1656-2 (CP001921.1)	3.94	222/56.35	3657/928	Hypoxanthine phosphorybosyl transferase	27	AGAAAAAAAGAGA **AAGAAGAGA** AAAAA	16 (0.0006)	48.2
						AGA **AAAAAAGAG** AAAGAAGAGAAAAAA	20 (2.32 e-05)	46.1
						AGA **AAAAAAGAGA** AAGAAGAGAAAAAA	20 (2.32 e-05)	46.1
						AGA **AAAAAAGAGA** AAGAAGAGAAAAAA	20 (2.32 e-05)	45.5
						AGA **AAAAAAGAGA** AAGAAGAGAAAAAA	20 (2.32 e-05)	45.5
				Intergenic	26	**AAAAAGAGGA** AAAAGAAAAAAAAGAA	18 (0.0001)	35.2
						AAA **AAGAGGAAAA** AGAAAAAAAAGAA	18 (0.0001)	40.9
				MiaA	25	AAAAAAAGGAAAAA **GAGGAGAAAGA**	18 (3.01e-005)	50.6
				ParC	23	AGAAAAAAGAAAAA **AAGAAAAGA**	17 (0.0002)	29.3
				Acetyl-coenzyme A carboxyl transferase	23	AA **AAAAGAAGA** AAAAAAAGAAAA	17 (0.0002)	30.2
				Putative acyl-CoA carboxylase alpha chain protein	21	AGAGAAAGAAAAAAAGAAGAA	None	
				UbiA	21	AGAAAAAGAGAA **AGAAAAAGG**	17 (0.0002)	38.1
						A **GAAAAAGA** GAAAGAAAAAGG	16 (0.0005)	31.9
				Putative viulence factor MviN family	21	**AAGAAAAA** AAGAAAAAAGAAG	16 (0.0005)	28.1
						AAGAAAAA **AAGAAAAA** AGAAG	16 (0.0005)	28.1
ACICU (CP000863.1)	3.90	59/15.13	965/247	Hemerythrin-like metal-binding domain-containing protein	29	AGAGAAAAAGAAAAGA **AGAAAGGAAA** GAA	18 (0.0001)	47.3
				Uncharacterized conserved protein	21	AAGAAAAAGGAAAGGAGAGGA	None	
AYE (CU459141.1)	3.94	63/15.99	1000/254	Putative adenylate or guanylate cyclase	22	AAAAAAAGAAAAA **AAGGAGGAA**	15 (0.0012)	33.6
				Putative hemerythrin like protein	21	**GAAGAAGAA** AAAGAGAAGAAG	16 (0.0005)	51.4
						GAAGAAGAAAAA **GAGAAGAAG**	17 (0.0002)	53.4
BJAB07104 (CP003846.1)	3.95	74/18.73	1206/305	Hemerythrin-like metal-binding domain-containing protein	29	AGAGAAAAAGAAAAGA **AGAAAGGAAA** GAA	18 (0.0001)	47.3
				Adenylate cyclase, family 3 (some protein containing HAMP domain)	22	AAAAAAAGAAAAA **AAGGAGGAA**	15 (0.0012)	33.6
				Putative unusual protein kinase	21	**GAAGAAGAA** AAAGAGAAGAAG	16 (0.0005)	51.4
						GAAGAAGAAAAA **GAGAAGAAG**	17 (0.0002)	51.4
BJAB0868 (CP003849.1)	3.91	74/18.93	1207/309	Hemerythrin	29	AGAGAAAAAGAAAAGA **AGAAAGGAAA** GAA	18 (0.0001)	47.3
				Intergenic	21	AAGAAAAAGGAAAGGAGAGGA	None	
BJAB0715 (CP003847.1)	4.00	76/19.00	1242/311	Hemerythrin	29	AGAGAAAAAGAAAAGA **AGAAAGGAAA** GAA	18 (0.0001)	47.3
				Intergenic	21	GAGAAAAAGGAAAGGAGAGGA	None	
MDR-ZJ06 (CP001937.1)	3.99	66/16.54	1078/270	Putatitive heme acquistion system receptor	29	AGAGAAAAAGAAAAGA **AGAAAGGAAA** GAA	18 (0.0001)	47.3
				Intergenic	21	AAGAAAAAGGAAAGGAGAGGA	None	
MDR-TJ (CP003500.1)	3.96	72/18.18	1154/291	Hypothetical protein	21	**GGGAAAAG** GAGGAGGGAAAAG	15 (0.0012)	51.5
				Putative unusual protein kinase	21	**GAAGAAGAA** AAAGAGAAGAAG	16 (0.0005)	51.4
						GAAGAAGAAAAA **GAGAAGAAG**	17 (0.0002)	51.4
TCDC-AB0715 (CP002522.2)	4.14	78/18.84	1272/307	Hypothetical protein	29	AGAGAAAAAGAAAAGA **AGAAAGGAAA** GAA	18 (0.0001)	47.3
				Intergenic	21	AAGAAAAAGGAAAG GAGAGGA	None	
TYTH-1 (CP003856.1)	3.96	71/17.93	1159/293	Hemerythrin-like metal-binding domain-containing protein	29	AGAGAAAAAGAAAAGA **AGAAAGGAAA** GAA	18 (0.0001)	47.9
				Intergenic	21	AAGAAAAAGGAAAGGAGAGGA	None	
ZW85-1 (CP006768.1)	3.76	57/15.16	912/243	Hypothetical protein	21	GGGAAAAGGAGGA **GGGAAAAG**	15 (0.0012)	51.5
				Protein kinase	21	*(GAAGAAGAA)*AAAGAGAAGAAG	16 (0.0005)	51.4
						GAAGAAGAAAAA **GAGAAGAAG**	17 (0.0002)	51.4
				Intergenic	21	AAAAAAAGAAAAAAGGAGGAA	None	

*PR, Purine Repeat; SC, Susceptible strain; MDR, Multi-drug resistant strain*.

* D_N_, N/Genome Size (Mb);

** *D_L_, L/Genome Size (Mb)*.

### Purine repeat search

In the present study, purine repeats (PRs) are defined as poly(purine) tracks of size, *n* ≥ 15, without any single interruption of pyrimidine, which is a strict definition of triplex forming sequences. To identify the PRs, a GUI (graphical user interface) based standalone Windows-based software entitled “***nTrackAnnotator***” (http://www.aiims.edu/images/depart/biochem/nTrackAnnotator.rar) (Singh and Rajeswari, 2017) was used. The *nTrackAnnotator* is developed by our group, which can be used for eukaryotic/prokaryotic genome sequences, including PRs. The *nTrackAnnotator* was developed in C# 4.0 object oriented programming language at. NET Framework 4.0 for Windows-platform specific to NCBI/Genome data format.

### Density of purine repeats

In order to characterize the repeats, we used two measures of density, the density in number (*D*_N_) and the density in length (*D*_L_) (Kendall, 1938) and are defined as:

$$\begin{array}{l} D_{\text{N}}\;=\text{ no}.\text{ of copies/size of genome }(\text{Mb})\\ D_{\text{L}}\;=\;[\text{size of repeat sequence }(\text{bp})\text{/size of genome }(\text{Mb})] \end{array}$$

Kendall τ rank test (Kendall, 1938) has been performed to find the association between *D*_*N*_ and *D*_*L*_. The Kendall rank correlation coefficient (τ) between the two variables with “n” number of observations is defined as:

τ=(number of concordant pairs)−(number of discordant pairs)n(n−1)2

### Intra-molecular triplex motif search

The intramolecular triplex motifs were identified using the *Biocondcutor package “Triplex*,” which is an R interface to the C implementation of a dynamic programming algorithm for triplex search (http://bioconductor.org/packages/release/bioc/html/triplex.html) (Hon et al., 2013). The R package “triplex” detected positions of subsequences in the PRs, where these sub-sequences were likely to fold into an intramolecular triplex. Further, the triplex-score and *p*-value of the triplex motifs were also calculated to find the probability of occurrence of detected triplexes in a random sequence and to find the quality of intra-molecular triplex. A higher score value indicates a higher quality triplex. Further, the Bioconductor R package was also used for the visualization of exact base-paring in two dimensions.

### Prediction of the stability of intra-molecular DNA triplex

The stability of any DNA-triplex measured in terms of the melting temperature (Tm) depends on various factors, such as the composition of the sequence of DNA-triplex, its concentration and pH. A rough prediction of the melting temperature (Tm) of DNA-triplex in the genome was determined using Roberts and Crothers empirical equations (equations i–iii) (Roberts and Crothers, 1996; Goñi et al., 2004):

(1) Tm=310×ΔH◦ΔH◦−ΔG◦−310×R×ln×(4TCP)

where *C*_TP_ is the concentration of DNA- triplex and R is gas constant.

The enthalpy (Δ*H*°) is evaluated (in kcal/mol) using equation (ii):

(2) $$\Delta H^{◦}=-4.9\left(\text{CC}\right)-8.9\left(\text{TC}+\text{CT}\right)-7.4\left(\text{TT}\right)$$

The Gibbs free energy (Δ*G*°) at 37°C was computed using equation (iii):

(3) $$\begin{array}{l} \Delta G_{37}^{◦}=-3.00(\text{C})-0.65(\text{T})+1.65(\text{CC})+6.0\\ \;+\;(\text{C})(\text{pH}-\text{5}.\text{0})[1.26-0.08(\text{CC})] \end{array}$$

where (X) and (Y) represents cytosine and thymine respectively in the triplex forming sequence.

## Results and discussion

The notion that genomic sequences are random in nature is far from truth; interestingly they are made up of systematically ordered and information rich patterns. These repeated sequence patterns such as poly(purine) repeats have been reported to be linked with genome functions and organization (Bucher and Yagil, 1991; Achaz et al., 2002; Gil and Latorre, 2012). Therefore, to understand the significance of intra-molecular triplex and its integration of genetic factors that might be responsible for *A. baumannii* infection and pathogenecity, we undertook a whole-genome sequence analysis of 14 *A. baumannii* strains to explore common features of their evolution and the diversity of mechanisms. Our goal was to (i) identify triplex forming purine repeat distribution in the susceptible and clinical isolates, (ii) demonstrate presence of stable intra-molecular triplexes, and (iii) find the impact of triplexes in the infection evolution of the resistance phenotype. A contiguous PR of at least 10 base pairs is required for the duplex acceptor and can form intra-molecular triplexes (Cheng and Van Dyke, 1993; Orson et al., 1999). Since triplex is formed by shorter PRs are not stable under physiological conditions and even single base pyrimidine interruptions are known to greatly destabilized DNA-triplexes (Orson et al., 1999), therefore, we performed the analysis of PR, *n* ≥ 15 present in SC and MDR strains of *A. baumannii*.

### Distribution and enrichment of PRs in *A. baumannii* strains

The distribution of PRs in both susceptible (SC) and multi-drug resistant (MDR) bacterial genomes is given in Table 1. The distribution of PRs in different strains of *A. baumannii* was found to be in general comparable to between SC and MDR isolates except MDR strain *AB1656-2* (frequency, N, in Table 1) where the frequency is approximately 3-folds higher. The larger PR length of 29 nucleotides was observed in eight bacterial strains (SC strain: *ATCC-17978;* MDR strains: *ACICU, BJAB07104, BJAB0868, BJAB0715, MDR-ZJ06, TCDC-AB0715, ZW85-1*). Majority of PRs were found in the range of 15–20 nucleotides length (Table 1). The frequency of PRs was observed to be proportional to the length of PRs in corresponding bacterial strain except in the MDR strains “*TYTH-1*” and “*MDR-TJ*.” The *TYTH-1* strains carry 71 PRs with 1159 nucleotides purine repeat length, however, “*MDR-TJ*” carry 72 PRs with 1154 nucleotides length. The observation that the highest PRs (*n* = 222) found in MDR strain “*AB1656-2*” indicates the possibility of greater frequency of mutations. The existence of such large density of PRs could be due to massive duplications of short PRs in *A. baumannii* and also indicates its biological effect on genomic functions.

After identifying the PRs, they were characterized by using two parameters of repeat density; D_N_ and D_L_. It was not surprising to find the positive correlation between D_N_ and D_L_ (τ = 0.956; *p* = 2.5034e-06; Kendall τ rank test). As this observation supports the report that the repeat sequences (like transposable elements) have high chance of duplications in the corresponding chromosomes (Coissac et al., 1997; Achaz et al., 2001). However, the biological interpretation of these measures may be quite different. The D_N_ is assimilated to the rate of amplification, which is a fine balance between duplication and deletion processes. While, the D_L_ which is a measure of redundancy tolerated by the chromosome can be extrapolated to the evolutionary changes (Coissac et al., 1997; Achaz et al., 2001, 2002). For example, the D_N_ in *ATCC-17978* is only 19.85 per Mb as compared to that of an MDR strain, *AB1656-2* is 56.35 per Mb. Similarly, the D_L_ in *ATCC-1798* is 325 per Mb vs. *AB1656-2* is 928 per Mb. It is clear from the observed high PR density (D_N_ and D_L_) in the MDR strain; *AB1656-2* in *A. baumannii* hints their critical role in genomic adaptation processes.

### Annotation and functional importance of potential PRs (≥ 20) in *A. baumannii* strains

A glance at Table 1 reveals that there are a minimum of 56 PRs found in any strain, SC or MDR, of *A. baumannii*. However, amongst such a large number of PRs (total of 1,106 in 14 strains), only 4% (37 PRs) were found to carry larger than 20 purines. As the stability of DNA-triplex formed by a PR is directly dependent on its length, we chose to characterize the localization of these 37 PRs which are discussed in sequel.

In principle purine repeats can form intra-molecular DNA triple helical structure; however, this may not be always conducive. Lexa et al. (2011) reported that there could be two possible reasons for purine repeats to form not so stable DNA-triplex. There are: (i) analogical to mismatches in DNA duplex, involving nucleotides in triplets that do not readily form Hoogsteen bonds; (ii) geometrically incompatible neighboring bases hindering proper alignment of strands for optimal hydrogen bonding and stacking (Lexa et al., 2011). Therefore, the “Bioconductor R package: triplex,” was used to detect strong intra-molecular triplex forming sequences within the identified potential PRs. Further, stability of DNA-triplex was quantitatively determined by melting temperature (Tm) of the intra-molecular triplexes that were calculated on the basis of Roberts and Crothers empirical equations (Roberts and Crothers, 1996). The rules were derived from van't Hoff analysis using a number of different DNA-triplexes tested at a variety of pH values and to predict the stability of DNA triplex on the basis of the sequence.

By applying the “Bioconductor R package: triplex,” to all the 37 potential PRs, eight sequences found not form intra-molecular DNA-triplex. However, there may be multiple independent intra-molecular DNA triplexes possible within a certain PR, for example, ABC1 family protein (PR of 21 purines) shows two such sequences (Table 1). The results also showed that all PRs were of pyrimidine motif, with an exception of PR present in the protein, hypoxanthine phosphorybosyl transferase, of MDR strain *AB1656-2*, was found to be forming intra-molecular triplex of both purine and pyrimidine motifs from the same sequence.

The rest of 29 PRs were further evaluated for their relative intra-molecular DNA-triplex forming stability using melting temperature (Tm). The results showed that a large number (19) of PRs were more stable (Tm ≥ 40°C) as compared to those which Tm about 30°C (Table 1). It is tempting to speculate that when the bacteria enters in the host (human), the DNA-triplexes with Tm about 30°C are not be stable and therefore dissociate as the physiological temperature of the body 37°C, is above its Tm. Therefore, intra-molecular DNA-triplexes ≥ 40°C present in 20 genes were considered to be stable (Table 1).

Majority of PRs were limited in the coding regions of the genome and only 10 were located in inter-genic regions (Table 1). It is interesting to note that *AB165-2* strain also contains maximum (8) repeats with purine tracks (≥20) as compared to that of rest of 13 strains (Table 1). Further, the purine repeat with maximum number of purines, 29, was found to be conserved (sequence identity: 100%; query coverage: 100%) as single copy in the eight *A. baumannii* strains. The significant observation is that the PR with 29 purines was restricted to only “hemerythrin” or “hemerythrin like metal binding proteins” (Table 1). In this context, it is important to note that hemerythrin and like proteins are known to be associated with various cellular functions including detoxification, transport, storage of iron and/or oxygen or a role as a sensory protein (Chenier et al., 2008; Eijkelkamp et al., 2011; Fiester and Actis, 2013). Iron is a stress factor that plays a critical role in the virulence of *A. baumannii* and the pathogenesis of human infections. Under iron stress conditions, hemerythrin and hemerythrin like proteins were reported to be donwregulated in *A. baumanni* (Eijkelkamp et al., 2011; Fiester and Actis, 2013). Considering the vital role of iron and iron associated proteins like hemerythrin, it appears that the presence of large purines could be one of the major reasons in inducing mutations and DNA triplex mediated downregulation of these proteins. It clearly indicates that there is some relation that exists between hemerythrin gene related iron utilization and DNA triplex formation in the *A. baumannii*, however, the mechanisms need to be explored. In addition to this, many enzymes were identified carrying potential PR such as hypoxanthine phosphorybosyl transferase, acetyl-coenzyme A carboxyl transferase, adenylate cyclase, protein kinases, and hypothetical proteins (Table 1) could also be affected. The functions of these enzymes are in general are crucial and therefore can directly or in-directly play role in survival of the bacteria.

More importantly, we found several virulence factor genes (parC, MviN family gene, VbiA, MiaA) carrying potential PRs located only in the MDR strains indicating further the possible functions in bacterial pathogenesis (Table 1). Among the several factors that affect the appearance and spread of acquired antibiotic resistance, the mutation frequency and the biological cost of resistance are of special importance (Björkholm et al., 2001; Hammerstrom et al., 2015). It is reported that mutations in parC (topoisomerase IV subunit C) gene contribute in antibiotic susceptibility and for this reason parC gene has been one of the drug targets for *A. baumanni* infections (Vila et al., 1995; Vakili et al., 2014). The other virulence factor MviN family gene, although its function is not elucidated but bioinformatics analysis indicates that MviN is an integral membrane protein and appeared to be a member of the MviN MATE (multi-antimicrobial extrusion) like super-family shown to function as drug/sodium antiporters (Omote et al., 2006). These proteins have also been shown to mediate resistance to a wide range of antibiotics drugs including fluoroquinolones, aminoglycosides etc. (Omote et al., 2006). Thus, the identification of potential PRs in the above mentioned genes in *A. baumannii* suggests their crucial role in infections, pathogenesis and drug-resistance either by triplex mediated mutatgenesis or gene deregulation.

## Conclusions

Based on the *in-silico* analysis of *A. baumannii* genome of clinical strains, the following major conclusions can be drawn. Triplex forming purine repeat sequences (PRs) were found to be randomly distributed (genic and intergenic) across the entire *A. baummannii* strains. The PRs, *n* ≥ 20, referred to as “Potential PRs.” Presence of potential PRs in bacterial genome reveal strong possibility to form stable intra-molecular DNA-triplex that can induce mutations and thereby interfere with genome functions in *A. baumannii*. As, iron plays important role in bacterial survival, the identification of “potential PRs” in genes of hemerythrin and hemerethyrin-like binding proteins, which are associated with iron utilization, is considered to be a significant finding of the present study. Equally important are the potential PR associated genes of enzymes (transferase, cyclase, kinase etc.) and of virulence factors (parC, UbiA, MiaA, MviN); which are known for their functional role in bacterial adaptation and infections.

## Author contributions

MR: designed the experiments; HS: performed the experiments and analyzed the data; MR: analyzed the data, drafted, edited, and approved the manuscript.

### Conflict of interest statement

The authors declare that the research was conducted in the absence of any commercial or financial relationships that could be construed as a potential conflict of interest.
